# Use of Insecticide-Treated School Uniforms for Prevention of Dengue in Schoolchildren: A Cost-Effectiveness Analysis

**DOI:** 10.1371/journal.pone.0108017

**Published:** 2014-09-23

**Authors:** Yesim Tozan, Pitcha Ratanawong, Valérie R. Louis, Pattamaporn Kittayapong, Annelies Wilder-Smith

**Affiliations:** 1 Institute of Public Health, Ruprecht-Karls-University, Heidelberg, Germany; 2 Steinhardt School of Culture, Education and Human Development, New York University, New York, New York, United States of America; 3 Center of Excellence for Vectors and Vector-Borne Diseases, Faculty of Science, Mahidol University at Salaya, Nakhon Phatom, Thailand; 4 Lee Kong Chian School of Medicine, Nanyang Technological University, Singapore, Singapore; 5 University of Umea, Umea, Sweden; Swiss Tropical & Public Health Institute, Switzerland

## Abstract

**Background:**

Dengue-related illness is a leading cause of hospitalization and death, particularly among children. Practical, acceptable and affordable measures are urgently needed to protect this age group. Schools where children spend most of their day is proposed as an ideal setting to implement preventive strategies against day-biting *Aedes* mosquitoes. The use of insecticide-treated school uniforms is a promising strategy currently under investigation.

**Methods:**

Using a decision-analytic model, we evaluated the cost-effectiveness of the use of insecticide-treated school uniforms for prevention of dengue, compared with a “do-nothing” alternative, in schoolchildren from the societal perspective. We explored how the potential economic value of the intervention varied under various scenarios of intervention effectiveness and cost, as well as dengue infection risk in school-aged children, using data specific to Thailand.

**Results:**

At an average dengue incidence rate of 5.8% per year in school-aged children, the intervention was cost-effective (ICER≤$16,440) in a variety of scenarios when the intervention cost per child was $5.3 or less and the intervention effectiveness was 50% or higher. In fact, the intervention was cost saving (ICER<0) in all scenarios in which the intervention cost per child was $2.9 or less per year and the intervention effectiveness was 50% or higher. The results suggested that this intervention would be of no interest to Thai policy makers when the intervention cost per child was $10.6 or higher per year regardless of intervention effectiveness (ICER>$16,440).

**Conclusions:**

Our results present the potential economic value of the use of insecticide-treated uniforms for prevention of dengue in schoolchildren in a typical dengue endemic setting and highlight the urgent need for additional research on this intervention.

## Introduction

Recent estimates put the global public health burden of dengue infections in 2010 at 390 million infections per year with 96 million symptomatic cases, affecting Southeast Asian countries disproportionately [1]. Among symptomatic cases, disease severity varies from mild, self-limiting febrile illness to severe to fatal hemorrhagic disease–the latter more commonly experienced by children and adolescences under the age of fifteen [2], [3]. There are currently no vaccines or specific antiviral drugs. Dengue-related illness is a leading cause of hospitalization, particularly among children [2]–[4], with case fatality rates of 1-5% among patients with dengue shock syndrome [5], placing heavy socio-economic burden on households and putting enormous pressure on strained health systems in endemic countries [4], [6], [7], particularly during outbreaks [8]–[11].

In the absence of vaccines and antiviral therapies, dengue prevention and control have relied heavily on vector control interventions that aim to reduce the population of dengue-carrying *Aedes* mosquitoes through the application of larvicides and adulticidal insecticide space sprays and management of breeding sites [12]. These community-based vector control efforts have, however, had limited impact on the increasing incidence and the geographic expansion of dengue in endemic countries and beyond [13]–[15]. Practical, acceptable and affordable measures are urgently needed, particularly to protect vulnerable children at risk of dengue infection. *Aedes* mosquitoes mainly bite during the day [16]. Because children spend most of their day at school, it has been suggested that preventive strategies should target schools and school activities [17]. Schoolchildren in most endemic countries wear school uniforms as a social norm [18]. A recent review on the safety and effectiveness of the use of insecticide-treated clothing indicated that it is a promising intervention, depending on the targeted vector and the pathogen transmission potential, and that studies demonstrated a wide range of effectiveness from nil to 79% in reducing disease incidence [19]. A randomized controlled trial is underway in Thailand to establish the effectiveness of insecticide-treated school uniforms for prevention of dengue in schoolchildren [20]. Further, permethrin-treated school uniforms are currently being tested under laboratory conditions to measure their knock-down efficacy under different types of treatments [19], [20]. A recent mathematical modeling study showed that the use of insecticide-treated school uniforms could potentially reduce the incidence of dengue infection up to 55% in schoolchildren, depending on a number of factors such as the proportion of mosquito bites received during school time, the probability that mosquitoes will come into contact with the insecticide, and the level of compliance among schoolchildren with the intervention [21].

Economic evaluation using decision analytical modeling makes it possible to assess the potential health and economic value of new health technologies in advance of randomized controlled trials. As a vehicle for economic evaluation, decision models can synthesize the available epidemiological, clinical, and economic evidence, examine multiple sources and consequences of uncertainty in the available evidence, and identify the parameters that have the greatest effect on the cost-effectiveness of new technologies. Results can guide further research on new technologies during development, or inform policy decisions about their adoption and use, particularly in resource-limited health care settings. Cost-effectiveness frameworks can be updated as new evidence on the safety, efficacy and effectiveness of new technologies and their costs becomes available from randomized trials, observational studies, and systematic reviews.

Using a decision-analytic model, we evaluated the cost-effectiveness of the use of insecticide-treated school uniforms for prevention of dengue in schoolchildren from the societal perspective. We explored how the potential economic value of the intervention varied under various scenarios of intervention effectiveness and cost, as well as dengue infection risk in school-aged children, using data specific to Thailand.

## Methods

Using TreeAge Pro 2014 (TreeAge Software Inc., Williamstown, MA, USA), we developed a decision-analytic model to simulate the decision on using insecticide-treated school uniforms for prevention of dengue in schoolchildren in an endemic setting. The decision model evaluated the expected costs and health outcomes of the intervention in a hypothetical cohort of schoolchildren. The intervention was then compared with a “do-nothing” alternative, following the standard guidelines of economic analyses [22]. The time horizon of the analysis is one year under a conservative assumption that children would require a new set of school uniforms each year. The outcome of the cost-effectiveness analysis was expressed as a ratio of incremental costs to incremental health outcomes–that is, incremental cost-effectiveness ratios (ICERs). Incremental health outcomes were estimated in terms of DF cases (non-hospitalized and hospitalized), DHF cases, and disability-adjusted life years (DALYs) averted. DALYs combine years of life lost because of premature death and years of life lived with disability in a single health outcome measure. ICERs were calculated as the cost per DALY averted. We reported ICERs when an alternative strategy was not ruled out of the decision analysis by simple dominance (i.e. less costly and more effective). Incremental costs and ICERs were calculated in United States (US) dollars ($) for the year 2012. All input parameters, their distributions, and data sources are listed in Table 1.

**Table 1:** Decision model parameters.

Parameter	Base value (SD or range)	Distribution	Source
Annual dengue incidence rate (%)	5.8	Beta (331, 5429)	[25]
Proportion of asymptomatic cases (%)	53.4	Beta (177, 154)	[25]
Proportion of non-hospitalized DF cases (%)	81.5	Point estimate	[25]
Proportion of hospitalized DF cases (%)	7.4	Point estimate	[25]
Proportion of hospitalized DHF cases (%)	11.1	Point estimate	[25]
Case fatality rate for DF (%)	0.0027	Point estimate	[31]
Case fatality rate for DHF (%)	0.15 (0.0002)	Beta	[32]
Duration of illness for non-hospitalized DF (days)	4.4 (1–25)	LogNormal (1.48, 0.09)	[26]
Duration of illness for hospitalized DF (days)	6.4 (2–17)	LogNormal (1.85, 0.11)	[26]
Duration of illness for DHF (days)	8.4 (3–25)	LogNormal (2.12, 0.09)	[26]
Duration of hospitalization for dengue (days)	4.9 (3.3)	LogNormal (1.59, 0.05)	[6]
Disability weight for symptomatic DF	0.197	Point estimate	[34]
Disability weight for DHF	0.545	Point estimate	[34]
Social discount rate	0.03	Point estimate	[35]
Effectiveness of insecticide-treated school uniforms (%)	5–100	Threshold analysis	−
	50 (low) 75 (high) 100 (full)	Scenario analysis	
Number of ambulatory visits for non-hospitalized dengue^a^	4.2 (2.7)	LogNormal (1.43, 0.02)	[6]
Number of ambulatory visits for hospitalized dengue	4.2 (2.0)	LogNormal (1.43, 0.04)	[6]
Number of school days lost for non-hospitalized dengue^a^	4.2 (3.2)	LogNormal (1.43, 0.02)	[6]
Number of school days lost for hospitalized dengue	5.5 (3.4)	LogNormal (1.70, 0.05)	[6]
Number of work days lost for non-hospitalized dengue^a^	4.0 (5.6)	LogNormal (1.39, 0.05)	[6]
Number of work days lost for hospitalized dengue^a^	3.9 (5.0)	LogNormal (1.36, 0.10)	[6]
Cost per ambulatory care visit^b^	15.87	Point estimate	[6]
Minimum daily wage^c^ (300 TBH)	9.81	Point estimate	[36]
Daily cost of providing education per student^b^	2.34	Point estimate	[6]
Cost of food for an attendant family member per day of hospitalization^c^ (50 THB)	1.63	Point estimate	[26]
Cost of transportation for an attendant family member per clinical visit or day of hospitalization^c^ (10 THB)	0.33	Point estimate	[26]
Mark-up cost of impregnation per child per year	0–10	Threshold analysis	−
	2.5 (low) 5 (moderate) 10 (high)	Scenario analysis	

(all costs are in US dollars for the year 2012).

DF = Dengue Fever; DHF = Dengue Hemorrhagic Fever; THB: Thai Baht.

a Values correspond to mean values reported for 8 dengue endemic countries [6].

b Costs reported for 2005 were adjusted to 2012, using an inflation rate of 3%.

c Using an average exchange rate of 1 USD  =  THB 30.60 for the year 2012 [36].

Following the recommendations of the World Health Organization (WHO) Commission on Macroeconomics and Health, we used the 2012 per head gross domestic product (GDP) for Thailand of $5,480 as a benchmark for intervention cost-effectiveness. The intervention was classified as highly cost-effective if the cost per DALY averted was less than the GDP per head ($5,480), and as cost-effective if this cost was less than one-to-three times the GDP per head ($5,480–$16,440). A summary measure of gross value added by all resident producers in a given country, the GDP per head reflects the fair share of residents to national economic output that can be devoted to health care. Given these cost-effectiveness benchmarks, the intervention would be unlikely to be considered by Thai policy-makers for implementation when the cost per DALY averted is higher than $16,440.

### Estimating health outcomes

The effectiveness of the intervention has not been established in real-life settings yet, but is expected to be around 50% [18]. This intervention cannot be 100% effective because school uniforms do not cover the entire body and are not worn all day long, during the weekends and school holidays. Children are not being bitten by *Aedes* mosquitoes only during school time. The knock-down effect of the insecticide on school uniforms does not reach 100% even under ideal laboratory conditions with currently used impregnation methods [21]. Lastly, there might also be compliance issues with the intervention in an everyday context [19]. In the absence of evidence on intervention effectiveness, we considered a very broad range of possible estimates of effectiveness from 5% to 100% to determine the threshold price points at which the intervention would be considered cost-effective given the cost-effectiveness benchmarks stated above. But we reported the results of the probabilistic cost-effectiveness analysis for intervention effectiveness 50% or higher. A public health intervention for prevention with less than 50% effectiveness (i.e. averting less than 50% of a disease) would be of no policy interest in resource-limited settings [23], [24], which set the effectiveness level at the low end for the analysis. Drawing on the range of possible effectiveness reported for insecticide-treated clothing in the literature [19], we assumed 75% effectiveness at the high end. The full effectiveness (100%) scenario was included to show the maximum potential benefit of the intervention for illustrative purposes. In the decision model, schoolchildren wearing insecticide-treated uniforms had a decreased risk of acquiring dengue infection by 1-intervention effectiveness. Acquiring dengue infection could result in asymptomatic infection or symptomatic infection, the latter requiring ambulatory care or hospitalization, followed by full recovery or death.

The clinical outcome probabilities used in the analysis were derived primarily from a multi-year prospective study on the epidemiology of acute dengue infection in a well-defined schoolchildren population in Thailand [25]. Over a period of three years, the overall incidence of dengue infection was 5.8%, and 54% of the infections were asymptomatic [25]. Of the symptomatic cases, the proportion of non-hospitalized dengue fever (DF), hospitalized DF, and hospitalized dengue hemorrhagic fever (DHF) were 81.5%, 7.4% and 11.1%, respectively [25]. These clinical outcome probabilities were used for the base case analysis.

The dengue disease burden (incidence, symptomatic cases, hospitalizations, deaths) varies from year to year with the circulating dengue virus strains. The prospective study in Thai schoolchildren reported that there were marked variations in the incidence of dengue infection and the spectrum of illness over the study period. This form of inter-annual variability in the clinical outcomes of dengue infections are unavoidable and may partially explain differences in parameter estimates across different endemic settings. It is not appropriate to use this type of variability, as opposed to other types of uncertainty, in the decision model to draw conclusions on the cost-effectiveness of the intervention. However, it is important to recognize the effect of inter-annual variability of dengue disease on the results. Therefore we examined the potential economic value of the intervention for a high incidence year and a low incidence year, with a dengue incidence rate of 7.9% and 2.2% per year, respectively [25]. During the high incidence year, 54% of the dengue infections were asymptomatic, and of the symptomatic cases, the proportion of non-hospitalized DF, hospitalized DF, and hospitalized DHF were 80.6%, 8.3% and 11.1%, respectively [25]. During the low incidence year, 63.6% of the infections were asymptomatic, and of the symptomatic cases, the proportion of non-hospitalized DF, hospitalized DF, and hospitalized DHF were 87.5%, 0% and 12.5%, respectively [25]. Lastly, the prospective study in Thai schoolchildren looked at the burden of symptomatic dengue infection and reported a mean duration of illness for non-hospitalized DF, hospitalized DF and hospitalized DHF of 4.4, 6.4 and 8.4 days, respectively [26].

The reported case fatality rates for the WHO Southeast Asia Region are around 1% [27], [28]. Focal outbreaks have, however, resulted in case fatality rates as high as 3–5% in some countries in the region [28]. It is possible to keep case fatality rates below 1% with early diagnosis and detection of shock [28]–[30]. Despite a steep increase in incidence and severity of dengue cases, Thailand has steadily reduced the case fatality rate of dengue to less than 0.2% over the past decade and pioneered the clinical management guidelines for DF and DHF [6], [29]. Based on the published literature, we used a case fatality rate of 0.0027% for DF patients (non-hospitalized or hospitalized) [31] and 0.15% for DHF patients [32]. DALY calculations considered an average life expectancy of 65.08 years based on the life tables for Thai men and women [33] and an average age of death of 10 years in this age group (5–15 years). We used a disability weight of 0.197 for DF and 0.545 for DHF [34]. DALYs were discounted at annual rate of 3%, as recommended by the World Bank [35]. Valuing a year of health life equally at all ages, we did not apply age weighting.

### Estimation of costs

Taking the societal perspective, we considered the direct cost of the intervention and the direct (medical and non-medical) and indirect costs of dengue-related illness over one year. The direct cost of the intervention included the cost of treating pre-fabricated school uniforms with a long-lasting insecticide (permethrin) formulation. Home- and factory-based impregnation methods are commonly used to treat clothing and nets used against vector-born diseases. New microencapsulation technologies, which may improve the residual activity of insecticides on clothing and reduce human exposure to insecticides, are on the horizon [19]. At the time of this analysis, the mark-up cost of insecticide treatment of pre-fabricated school uniforms was unknown in dengue endemic settings. In the randomized control trial in Thailand, the insecticide treatment cost of pre-fabricated school uniforms at an overseas factory using a factory proprietary method was about $5.50 per child, excluding the costs of international shipment and of collection of uniforms from and their distribution to families (Pattamaporn Kittayapong, personal communication). Therefore we ranged the mark-up cost of insecticide treatment of school uniforms between $0–10 per child per year in the threshold analysis and estimated the price points at which the intervention would be cost-effective across different levels of intervention effectiveness using the base values of the other parameters as presented in Table 1.

The published literature provides a detailed account of the economic burden of dengue-related illness on households and health systems. Most of the economic data used in this analysis were derived from a dengue cost-of-illness study in Asian and Latin American countries [6]. We considered the direct medical and non-medical costs of treatment for both ambulatory and hospitalized dengue patients. The cost-of-illness study reported that ambulatory and hospitalized patients averaged 4.2 ambulatory care visits during an illness episode, at an average cost of $15.87 per visit [6]. The costs of hospitalization were estimated by multiplying the mean duration of hospitalization with the cost per inpatient bed day. The mean duration of hospitalization for dengue was 4.9 days at a mean cost of $105.77 per inpatient bed day in Thailand [6]. We also estimated the direct non-medical costs associated with seeking care for dengue-related illness, such as food and transportation costs, and made the same unit cost assumptions with the prospective study in Thai schoolchildren [26]. We assumed that transport would be required for all ambulatory care visits and hospitalizations, and food costs would accrue for each day of hospitalization.

The published literature indicates that dengue-related illness negatively affects school attendance and productivity of household members. We estimated the indirect costs of ambulatory and hospitalized dengue cases. The cost-of-illness study reported that non-hospitalized and hospitalized dengue patients lost on average 4.2 and 5.5 school days, respectively [6]. The economic value of school absences was estimated as the product of the number of school days lost because of dengue-related illness and the daily per capita cost of providing education, which was estimated at $2.34 in Thailand [6]. Household members lost on average 4.0 and 3.9 workdays to care for non-hospitalized and hospitalized dengue patients, respectively. The economic value of lost earnings was conservatively estimated as the product of the average number of workdays lost because of dengue-related illness by the minimum daily wage of $9.81 in Thailand [36]. We excluded the economic value of premature deaths due to inherent difficulty of placing an economic value on death.

### Sensitivity analysis

Using scenario analysis, we examined the effects of two most critical parameters, namely intervention effectiveness and intervention cost per child per year, on the potential economic value of the intervention and determined threshold price points at which the intervention would cease to be cost-saving, highly cost-effective or cost-effective, using the base case values presented in Table 1. We also examined how the cost-effectiveness of the intervention varied with another critical parameter, annual dengue incidence rate, to recognize the effect of inter-annual variability in the clinical outcomes of dengue infections on the model outputs. All parameter estimates used to populate the model were obtained from published secondary sources. We performed univariate sensitivity analyses to examine the effect on the ICER of each parameter within its reported range (table 1). To represent uncertainty in parameter estimates obtained from secondary sources, distributions were chosen and fitted to reflect sampling uncertainty associated with their estimation by following standard approaches to distributional assumptions in health economic evaluation [37]. To test the robustness of the cost-effectiveness results, we performed probabilistic sensitivity analyses using a Monte Carlo simulation method where parameters were randomly sampled from their distributions (table 1). We considered three scenarios for intervention effectiveness– low effectiveness at 50%; high effectiveness at 75%; and full effectiveness at 100%– and three scenarios for the mark-up cost of insecticide treatment of pre-fabricated school uniforms per child– a low cost of $2.5, a moderate cost of $5, and a high cost of $10 per year. The results of the probabilistic cost-effectiveness analysis are reported under each of these scenarios.

## Results

**Figure 1:**
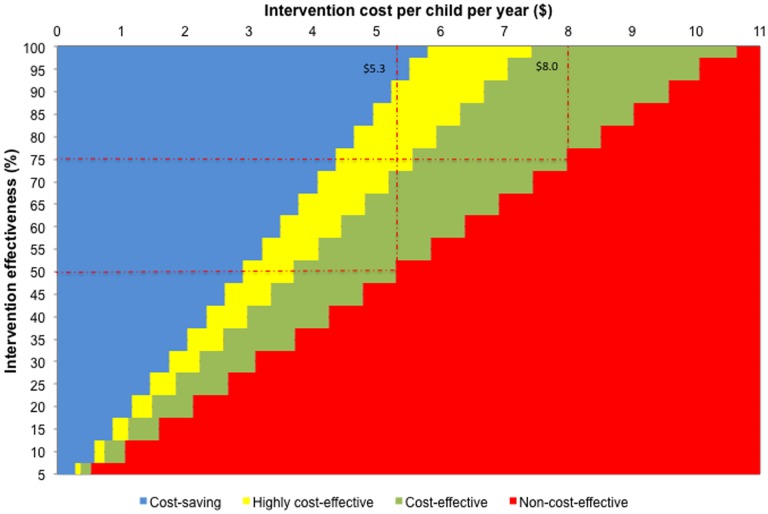
Threshold price points for the use of insecticide-treated uniforms for prevention of dengue in schoolchildren^a^ (all costs are in US dollars for the year 2012). ^a^ Using Thailand's GDP per capita of $5,480 as a threshold value, cost-saving, ICER <0; highly cost-effective, ICER <$5,480; cost-effective, $5,480 ≤ ICER ≤ $16,440; non-cost-effective, ICER> $16,440.

In the base case analysis, the cost-effectiveness of the intervention was assessed from the societal perspective over one year across varying levels of intervention effectiveness and costs at an average dengue incidence rate of 5.8% per year in school-aged children. Figure 1 presents the threshold price points for the intervention. We found the intervention to be cost-effective (ICER≤$16,440) in all scenarios in which intervention cost per child was $5.3 or less per year and intervention effectiveness was 50% or higher. In fact, the intervention was cost saving (ICER<0) in all scenarios in which intervention cost per child was $2.9 or less per year at intervention effectiveness 50% or higher. The intervention proved to be not cost-effective (ICER>$16,440) in scenarios in which intervention cost per child was more than $5.3 per year and intervention effectiveness was less than 50%. In general, the intervention would be of no interest to policy makers if intervention cost per child was $10.6 or higher per year.

Table 2 presents the results of the probabilistic cost-effectiveness analysis for the base case scenario. This table shows how the number of DF (non-hospitalized and hospitalized) and DHF cases averted varied by intervention effectiveness over one year. Under the full (100%) effectiveness scenario, the intervention would have averted on average 22 (95% CI, 19-25) cases of non-hospitalized DF, 2 (95% CI, 2-2) cases of hospitalized DF, and 3 (95% CI, 3-3) cases of DHF in a cohort of 1,000 schoolchildren over one year. At 50% effectiveness, 11 (95% CI, 9-13) cases of non-hospitalized DF, 1 (95% CI, 1-1) hospitalized DF, and 2 (95% CI, 1-2) cases of DHF would have been averted in the same cohort over the same period. Table 2 also shows that the cost of averting a DF or DHF case increased with increasing intervention cost at any given intervention effectiveness, and decreased with increasing intervention effectiveness at any given intervention cost. Cost-offsets included costs of prevented health care utilization related to treatment of dengue illness and costs of prevented productivity losses and school absences, and increased with increasing intervention effectiveness. In scenarios in which intervention effectiveness was 50% or higher and intervention cost is $2.9 or less per child, cost savings were associated with averting a case of DF or DHF, and the intervention was economically dominant over no intervention alternative. At a moderate intervention cost of $5 per child per year, per-case costs of averting non-hospitalized DF, hospitalized DF, and DHF could be as high as $198 (95% CI, 130-208), $2,182 (95% CI, 1,422-3,064), and $1,442 (95% CI, 986-2,018), respectively, while the intervention remained cost-effective for intervention effectiveness between 50%–100%.

**Table 2:** Results of the probabilistic cost-effectiveness analysis for the base case scenario with an annual dengue incidence rate of 5.8%.

Intervention effectiveness	Number of non-hospitalized DF cases averted^a^ (95% CI)	Cost per non-hospitalized DF cases averted^c^ (95% CI)	Number of hospitalized DF cases averted^a^ (95% CI)	Cost per hospitalized DF cases averted^c^ (95% CI)	Number of DHF cases averted^a^ (95% CI)	Cost per DHF case averted^b^ (95% CI)	Number of DALYs averted^a^ (95% CI)	Cost per DALY averted^c^ (ICER) (95% CI)
Intervention cost: $2.5 per child per year								
50%	11 (9−13)	−33 (−69−9)	1 (1−1)	−364 (−766 – 86)	2 (1−2)	−275 (−507−65)	0.14 (0.12−0.18)	***Dominant***
75%	16 (14−19)	−110 (−136−−81)	2 (1−2)	−1,212 (−1,487−−896)	2 (2−3)	−808 (−1,005−−601)	0.22 (0.18−0.27)	***Dominant***
100%	22 (19−25)	−148 (−170−−125)	2 (2−2)	−1,634 (−1,872−−1,388)	3 (3−3)	−1,089 (−1,248−−930)	0.29 (0.24−0.35)	***Dominant***
Intervention cost: $5 per child per year								
50%	11 (9−13)	198 (130−278)	1 (1−1)	2,182 (1,422 – 3,064)	2 (1 – 2)	1,442 (986 – 2,018)	0.14 (0.12 – 0.18)	***14,934 (9,662 – 21,498)***
75%	16 (14 – 19)	44 (−3 – 97)	2 (1 – 2)	484 (−22 – 1,068)	2 (2−3)	319 (−20−695)	0.22 (0.17−0.27)	***3,383 (−319 – 7,520)***
100%	22 (19−25)	−33 (−69−9)	2 (2−3)	−364 (−756 – 88)	3 (3−3)	−243 (−501−31)	0.29 (0.24−0.35)	***Dominant***
Intervention cost: $10 per child per year								
50%	11 (9−13)	659 (524−815)	1 (1−1)	7,285 (5,812 – 9,027)	2 (1−2)	4,879 (3,874 – 6,012)	0.15 (0.12−0.21)	49,480 (37,759 – 63,970)
75%	16 (14−19)	353 (262−457)	2 (1−2)	3,877 (2,887 – 5,029)	2 (2−3)	2,591 (2,919 – 3,335)	0.22 (0.17−0.27)	26,782 (19,342 – 35,487)
100%	22 (19−25)	198 (129−276)	2 (2−2)	2,188 (1,428 – 3,047)	3 (3−3)	1,455 (981 – 2,032)	0.29 (0.23 – 0.36)	***14,937 (9.278 – 21,100***)

(all costs are in US dollars for the year 2012).

DF = Dengue Fever; DHF = Dengue Hemorrhagic Fever; DALY = Disability-Adjusted Life Years; ICER = Incremental Cost-Effectiveness Ratio.

a Incremental health outcomes are presented per 1,000 children over one year.

b Negative cost values indicate cost-savings.

c Boldface font indicates scenarios that were cost-effective (ICER≤$16,440). Underlined entries in boldface font are scenarios in which the intervention was the dominant strategy (i.e. less costly and more effective than no intervention alternative).

**Figure 2:**
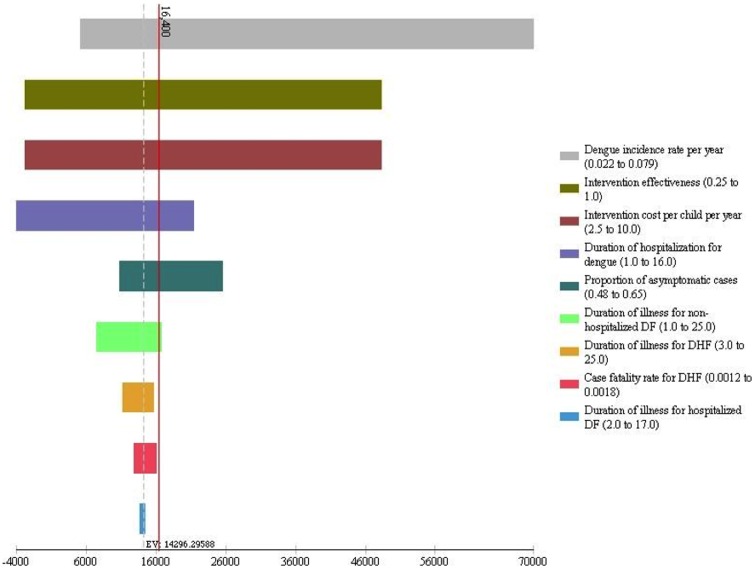
Tornado diagram of univariate sensitivity analyses. DF = Dengue Fever; DHF = Dengue Hemorrhagic Fever.

The results of the univariate sensitivity analyses are presented in a tornedo diagram in Figure 2. In the tornedo diagram, each bar represents the impact of variation in an individual parameter on the ICER and parameters are arranged from top to bottom in the order of their impact so that comparisons can be done visually. The solid vertical line on the diagram marks the ICER at an intervention cost of $5 and intervention effectiveness of 50% for the base case analysis. Horizontal bars to the left of this line indicate when the intervention is cost-effective, and bars to the right when it is not cost-effective. The results show that ICERs are most sensitive to dengue incidence rate, intervention effectiveness, and intervention cost, followed by duration of hospitalization for dengue and proportion of asymptomatic infections. This is because these parameters directly affect the avertable disease burden of dengue, modulating the incremental costs.

Table 3 presents the results of the probabilistic cost-effectiveness analysis for a low and a high dengue incidence year. The results are presented across varying levels of intervention effectiveness and costs similar to the base case analysis. In a low incidence year, the intervention was non-cost-effective (ICER>$16,440) in all scenarios except when the intervention cost per child was $2.5 or less per year and the intervention effectiveness was 60% or higher. In a high incidence year, the intervention was the economically dominant strategy (ICER<0) in most scenarios in which the intervention cost per child was $5 or less per year regardless of intervention effectiveness. The intervention proved to be cost-effective (ICER≤$16.440) even at a high intervention cost of $10 per year when intervention effectiveness was 70% or higher.

**Table 3:** Incremental cost-effectiveness ratios (ICERs) for low and high dengue incidence years^a^.

	Intervention cost per child per year					
	Annual dengue incidence rate: 2.2%			Annual dengue incidence rate: 7.9%		
Intervention effectiveness	$2.5	$5	$10	$2.5	$5	$10
50%	25,301 (21,620−29,207)	70,638 (60,934−81,071)	161,522 (140,176−185,879)	***Dominant***	***5,248 (4,039 – 6,506)***	30,587 (26,415 – 35,843)
75%	***10,190 (8,565−11,995)***	40,550 (34,818−47,129)	101,056 (86,536−116,880)	***Dominant***	***Dominant***	***13,637 (11,721 – 15,744)***
100%	***2,639 (1,620−3,664)***	25,281 (21,913−29,394)	70,742 (60,712−81,514)	***Dominant***	***Dominant***	***5,251 (3,941 – 6,573)***

(all ICERs are in US $ per DALY averted for the year 2012).

a Values represent ICERs expressed as cost per DALY averted. Boldface font indicates scenarios that were cost-effective (ICER≤$16,440). Underlined entries in boldface font are scenarios in which the intervention was the dominant strategy (i.e. less costly and more effective than no intervention alternative).

## Discussion

The use of insecticide-treated school uniforms for prevention of dengue in schoolchildren is a promising intervention given the existing body of evidence on the effectiveness of insecticide-treated clothing against vector-born diseases [19]. This modeling-based cost-effectiveness analysis examined the economic value of the intervention in a typical dengue endemic setting from the societal perspective. Cost-effectiveness is only one criterion to assess the merit of an intervention. Although insecticide treated clothing has been used by the military and can be found in recreational markets for personal protection for many years [19], the success of the intervention depends ultimately on the acceptability of insecticide-treated school uniforms by parents and the user compliance rate with the intervention, which would require extensive community mobilization efforts. A limited number of intervention trials reported greater acceptability and higher compliance rates if treated clothing or materials were personal items [19], [38], [39]. A recent study that took place in the context of the randomized clinical trial showed that the acceptability of the insecticide-treated school uniforms was high among parents, teachers, school principals, reflected by the lack of concern about and willingness to pay for and recommend them [40]. Further studies are needed to monitor the residual activity of insecticides on clothing over time to ensure its operational practicality in real world settings and to establish its long-term safety [19].

Using scenario analysis, we determined the threshold price points for the mark-up cost of insecticide treatment on school uniforms across different levels of intervention effectiveness at which their use would be cost-effective for prevention of dengue in schoolchildren (figure 1). At 50% effectiveness, the intervention cost per child should not exceed $5.3 per year. The threshold price point went up to $8 per child per year at 75% effectiveness. A major source of uncertainty in this analysis is the inter-annual variability of dengue disease patterns. While we showed its impact on the ICERs (table 3), our base case analysis using an average dengue incidence rate of 5.8% per year showed that the intervention could be cost-effective, even cost-saving, under a variety of scenarios (figure 1 and table 2).

Our cost-effectiveness framework can be easily updated as new evidence, particularly on the efficacy, safety, acceptability and cost of the intervention, becomes available from randomized trials, observational studies and systematic reviews. The framework can also be adapted to other dengue endemic settings to inform local decision-making; but policymakers should always contextualize costs and cost-effectiveness benchmarks and assess local disease and health care utilization patterns and other key parameters in their own settings to arrive at more locally representative ICERs. Nevertheless, cost-effectiveness does not necessarily indicate affordability, particularly in developing country settings where health budgets are constrained. The budget impact of the adoption of a new intervention in a specific setting is an important decision criterion for priority-setting in health care along with effectiveness, cost-effectiveness, and severity of illness. Commissioned by the government agency which manages the largest health plan in Thailand, a recent study described the evaluation process of a set of interventions to be included in the universal health coverage benefit package and defined a low budget impact intervention as one that would cost THB 200 million per annum or less to implement [24]. There are currently about 8 million schoolchildren (6–14 years) in Thailand [41]. Based on this budget impact criterion, the use of insecticide-treated school uniforms for prevention of dengue among schoolchildren would not be a candidate for public reimbursement if the mark-up cost of insecticide treatment of school uniforms is more than $1 per child per year.

Increasing severity and frequency of dengue outbreaks and the geographic expansion of dengue transmission within endemic countries pose a serious challenge to vulnerable populations. Children carry the brunt of the disease burden of dengue with high case fatality rates. Previous research has been inconclusive on whether children are more frequently contracting dengue at school or home [25], [42]–[44]. Nevertheless, current prevention programs that mainly target residential areas have had limited impact on the increasing burden of dengue illness. Strategies to protect children against dengue infection at schools remain a neglected research area [43]. There have been calls for practical, acceptable and affordable preventive strategies to protect children living in dengue endemic areas. As we wait for the development of an effective vaccine in the fight against dengue, this model-based economic evaluation study indicates the potential economic value of a newly proposed preventive strategy and highlights the need for additional research on the intervention to policy makers, manufacturers, researchers and other key stakeholders.

## Conclusions

Current dengue control efforts failed to address the increasing disease and economic burden of dengue in endemic countries. Practical, acceptable and affordable preventive strategies are needed to protect children at risk of dengue infection. Our results present the potential economic value of the use of insecticide-treated uniforms for prevention of dengue in school children in a typical dengue endemic setting and highlight the urgent need for additional research on this intervention.
