# Correction: Use of Insecticide-Treated School Uniforms for Prevention of Dengue in Schoolchildren: A Cost-Effectiveness Analysis

**DOI:** 10.1371/journal.pone.0118038

**Published:** 2015-02-03

**Authors:** The PLOS ONE Staff

References 32, 33, 36, and 41 are incomplete. Please view the corrected references here.

32. WHO SEARO. [cited 2014 January 3]; Available from: http://209.61.208.233/LinkFiles/Dengue_CFR_Dengue_85-06.pdf.

33. WHO Global Health Observatory Data Repository: Life expectancy. [cited 2014 January 14]; Available from: http://apps.who.int/gho/data/node.main.692.

36. Office of the Prime Minister In: Thailand, editor. Thailand. [cited 2014 February 11]; Available from: http://thailand.prd.go.th/view_news.php?id=6594&a=2.

41. UNESCO Institute for Statistics. [cited 2014 March 20]; Available from: http://www.uis.unesco.org/DataCentre/Pages/country-profile.aspx?code=THA&regioncode=40515

## References

[1] Tozan Y, Ratanawong P, Louis VR, Kittayapong P, Wilder-Smith A (2014) Use of Insecticide-Treated School Uniforms for Prevention of Dengue in Schoolchildren: A Cost-Effectiveness Analysis. PLoS ONE 9(9): e108017 doi: 10.1371/journal.pone.0108017 25247556 10.1371/journal.pone.0108017 PMC4172602

